# GSG2 (Haspin) promotes development and progression of bladder cancer through targeting KIF15 (Kinase-12)

**DOI:** 10.18632/aging.103005

**Published:** 2020-05-21

**Authors:** Yuhao Chen, Dian Fu, Hai Zhao, Wen Cheng, Feng Xu

**Affiliations:** 1 Department of Urology, Jinling Hospital, Medical School of Nanjing University, Nanjing 210002, Jiangsu, China

**Keywords:** bladder cancer, GSG2, KIF15, RNA sequencing, tumor promotor

## Abstract

Bladder cancer is the most commonly diagnosed malignant tumor in urological system worldwide. The relationship between GSG2 and bladder cancer has not been demonstrated and remains unclear. In this study, it was demonstrated that GSG2 was up-regulated in bladder cancer tissues compared with the normal tissues and its high expression was correlated with more advanced malignant grade and lower survival rate. Further investigations indicated that the overexpression/knockdown of GSG2 could promote/inhibit proliferation, colony formation and migration of bladder cancer cells, while inhibiting/promoting cell apoptosis. Moreover, knockdown of GSG2 could also suppress tumorigenicity of bladder cancer cells *in vivo*. RNA-sequencing followed by Ingenuity pathway analysis (IPA) was performed for exploring downstream of GSG2 and identified KIF15 as the potential target. Furthermore, our study revealed that knockdown of KIF15 could inhibit development of bladder cancer *in vitro*, and alleviate the GSG2 overexpression induced promotion of bladder cancer. In conclusion, our study showed, as the first time, GSG2 as a prognostic indicator and tumor promotor for bladder cancer, whose function was carried out probably through the regulation of KIF15.

## INTRODUCTION

Bladder cancer possesses the highest incidence among the malignant tumors of urinary system in China, and its incidence rate ranks ninth among all malignant tumors worldwide [1–3]. Among all cases of patients with tumor, bladder cancer accounts for 7% and 2% in men and women, respectively [3]. In new cases of bladder cancer, about 75% of patients had tumors confined to the bladder, while another 25% suffered from regional lymph node metastases or distant metastases at the time of diagnosis [4]. At present, the treatment strategies of bladder cancer mainly include surgical treatment, radiation treatment, chemotherapy and biological treatment [5]. With the rapid development of tumor genomics and tumor molecular biology research, a large number of studies have dramatically improved people's understanding of the occurrence and development of bladder cancer [6]. Sequentially, molecular targeted therapy has gradually become a hot spot in the field of tumor research. Although existing studies have identified some oncogenes or tumor suppressor genes that play important roles in the occurrence and development of bladder cancer, the treatment efficacy and prognosis of bladder cancer have not been satisfactorily improved [5, 7]. Therefore, in-depth research to clarify the molecular regulatory network of bladder cancer is of great significance for the development of bladder cancer targeted drugs to improve the prognosis.

Haploid germ cell-specific nuclear protein kinase (Haspin, alias GSG2) is an atypical eukaryotic protein kinase that was for a long time considered an inactive pseudokinase due to low degree of structural homology of GSG2 with the 'classical' protein kinases [8, 9]. GSG2 is a serine/threonine kinase that associates with chromosome and phosphorylates threonine 3 of histone 3 during mitosis [10]. Recently, GSG2 has been identified as a promising target for the design of inhibitors as potential anticancer drugs with relatively lower side-effects because of that threonine 3 of histone 3 is the only substrate of GSG2 [10, 11]. For example, Han et al. found that GSG2 can be considered as a viable anti-melanoma target, and the concomitant inhibition of GSG2 could act as a novel therapeutic target with improved efficacy for treatment of melanoma [12]. On the other hand, although it has been shown that GSG2 overexpression could result in defective mitosis of cells, thus regulating proliferation of cancer cells, the role of GSG2 in human cancers is still not clear. More importantly, the relationship between GSG2 and bladder cancer has never been studied and remained unknown.

Bearing all these in mind, the purpose of this study is to reveal the role of GSG2 in the development and progression of bladder cancer. Our results indicated the upregulation of GSG2 in tumor tissues and cell lines of bladder cancer in comparison with normal tissues and cell line, which was associated with malignant grade and prognosis of patients. Deficiency of GSG2 could inhibit bladder cancer development *in vitro* or *in vivo* through inducing the restrain of cell growth and the promotion of cell apoptosis and cell cycle arrest. Additionally, the investigation of regulatory mechanism of GSG2 on bladder cancer identified KIF15 as a potential downstream of GSG2.

## RESULTS

### GSG2 was up-regulated in bladder cancer and associated with poor prognosis

**Figure 1:**
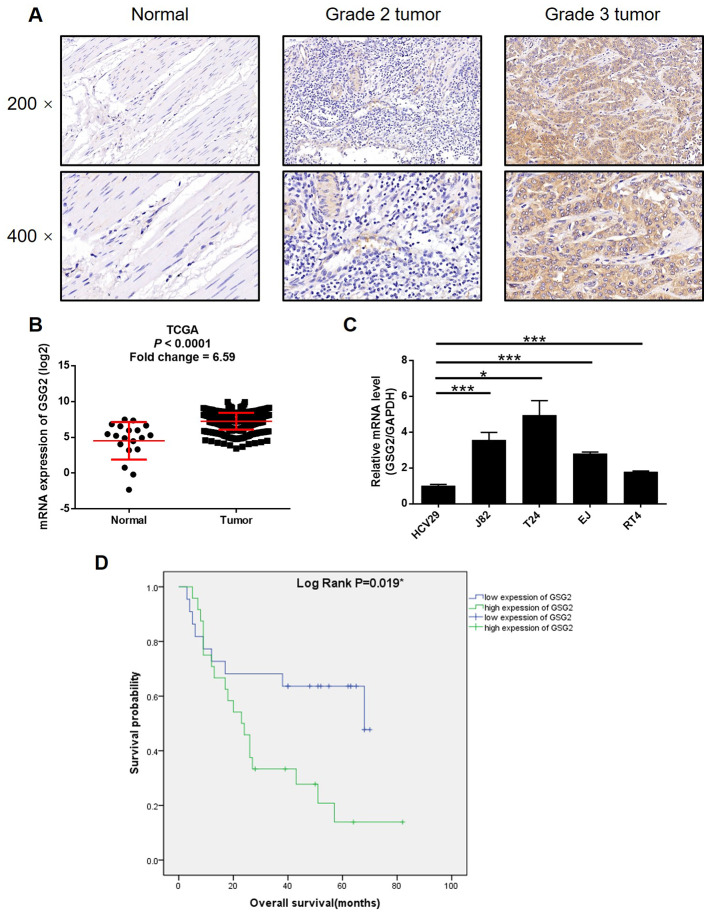
**GSG2 was up-regulated in bladder cancer.** (**A**) The expression of GSG2 in bladder cancer tissues and normal tissues was detected by IHC. (**B**) Data mining of TCGA database showed that expression of GSG2 is relatively higher in bladder cancer tissues compared with normal tissues. (**C**) Endogenous expression of GSG2 in human bladder epithelial cell line HCV29 and bladder cancer cell lines including RT4, EJ, T24 and J82 was detected by qPCR. (**D**) Kaplan-Meier survival analysis was performed to reveal the relationship between GSG2 expression and prognosis of bladder cancer patients. The figures are representative data from at least three independent experiments. The data were expressed as mean ± SD (n ≥ 3), *P<0.05, **P<0.01, ***P<0.001.

First, immunohistochemistry analysis and western blotting were performed to visualize the expression of GSG2 in clinical specimens collected from bladder cancer patients. It could be observed that GSG2 expression was remarkably higher in bladder cancer tissues than corresponding normal tissues (Figure 1A, Supplementary Figure 1A, and Table 1). Moreover, as shown by the representative tumor samples with different malignant grade, the expression of GSG2 increase along with the elevation of malignant grade, which was further confirmed by the statistical analysis based on GSG2 expression and the tumor characteristics of all 56 patients included in this experiments (Figure 1A, Supplementary Figure 1A and Table 2, Supplementary Table 1). Meanwhile, we also checked the expression profile of GSG2 in bladder cancer tissues and normal tissues in The Cancer Genome Atlas (TCGA), which was in agreement with our abovementioned results (Figure 1B). Similarly, it was also demonstrated that the expression of bladder cancer cell lines, including J82, T24, EJ and RT4, was significantly higher than normal bladder epithelial cell line HCV29 (Figure 1C). On the other hand, Kaplan-Meier survival analysis showed that patients with relatively higher expression of GSG2 suffered from shorter survival period (Figure 1D). These results suggested the probable involvement of GSG2 in the development and progression of bladder cancer.

**Table 1:** Expression patterns of GSG2 in bladder cancer tissues and normal tissues revealed in immunohistochemistry analysis.

**GSG2 expression**	**Tumor tissue**		**Normal tissue**	
	**Cases**	**Percentage**	**Cases**	**Percentage**
Low	26	46.4%	43	100%
High	30	53.6%	0	-

*P* < 0.001

**Table 2:** Relationship between GSG2 expression and tumor characteristics in patients with bladder cancer.

**Features**	**No. of patients**	**GSG2 expression**		***P* value**
		**low**	**high**	
All patients	56	26	30	
Age (years)				0.776
≤71	29	14	15	
>71	27	12	15	
Gender				0.394
Male	47	23	24	
Female	9	3	6	
Tumor size				0.613
<4 cm	23	12	11	
≥4 cm	31	14	17	
Lymphadenopathy				0.495
yes	6	2	4	
no	35	17	18	
Grade				0.003**
2	17	13	4	
3	39	13	26	
Stage				0.813
I	6	3	3	
II	10	5	5	
III	16	8	8	
IV	7	3	4	
T Infiltrate				0.857
T1	10	5	5	
T2	15	8	7	
T3	21	9	12	
T4	3	2	1	

### GSG2 knockdown regulated proliferation, apoptosis and migration of bladder cancer cells

**Figure 2:**
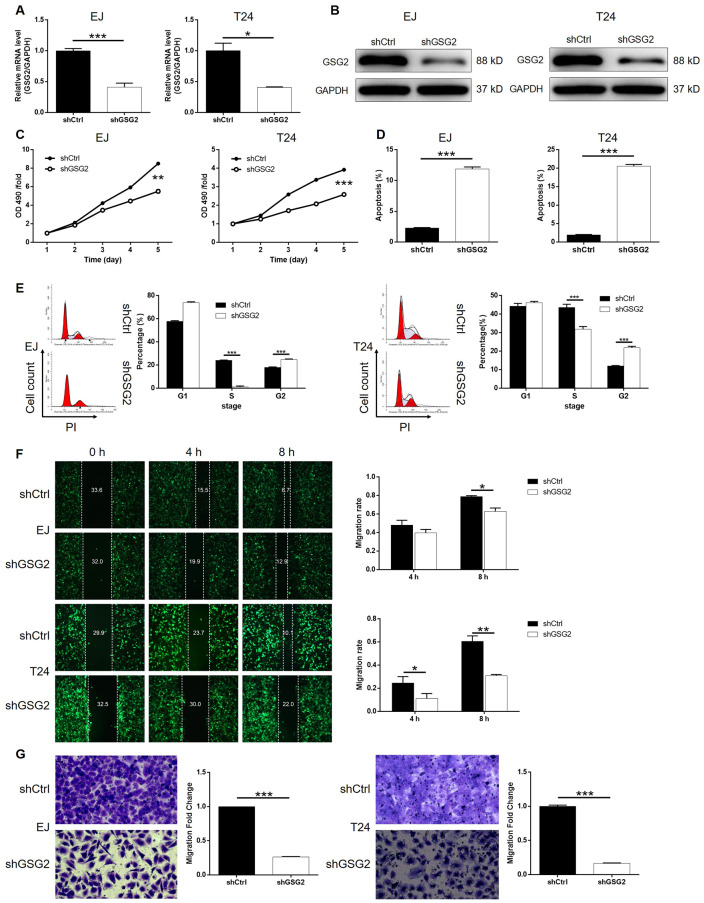
**GSG2 knockdown inhibited bladder cancer cell proliferation, cell migration and induced cell apoptosis and cycle.** (**A**) The knockdown efficiency of GSG2 in EJ and T24 cells was detected by qPCR. (**B**) The knockdown of GSG2 in EJ and T24 cells was verified by western blotting. (**C**) MTT assay was used to detect the effects of GSG2 knockdown on cell proliferation of EJ and T24 cells. (**D**) Flow cytometry was performed to evaluate the effects of GSG2 on cell apoptosis of EJ and T24 cells. (**E**) The effects of GSG2 knockdown on cell cycle distribution of EJ and T24 cells were examined by flow cytometry. Wound-healing (**F**) and Transwell (**G**) assays were utilized to reveal the effects of GSG2 knockdown on cell migration of EJ and T24 cells. The figures are representative data from at least three independent experiments. The data were expressed as mean ± SD (n ≥ 3), *P<0.05, **P<0.01, ***P<0.001.

For the sake of conducting a loss-of-function investigation of GSG2 on bladder cancer, lentivirus plasmids expressing shRNAs targeting GSG2 were prepared to transfect human bladder cancer cell lines EJ and T24 for silencing endogenous GSG2 expression. The successful construction of GSG2 knockdown cell lines was confirmed by highly efficient transfection (> 80%) (Supplementary Figure 1B), which was observed by fluorescence imaging, and significantly downregulation of GSG2 mRNA (P < 0.001 for EJ, P < 0.05 for T24 cells, Figure 2A) and protein levels (Figure 2B), which was obtained by qPCR and western blotting, respectively. The detection of cell viability in 5 continuous days by MTT showed that GSG2 knockdown induced remarkably suppression on cell proliferation (P < 0.01 for EJ, P < 0.001 for T24 cells, Figure 2C). The results of flow cytometry suggested that the inhibited cell growth by GSG2 knockdown may derive from the increased apoptotic cell proportion in shGSG2 group of cells (P < 0.001, Figure 2D). In order to preliminarily study the mechanism, a human apoptosis antibody array was used to identify differentially expressed proteins in shCtrl and shGSG2 T24 cells. The results demonstrated the downregulation of anti-apoptosis proteins including cIAP-2, HSP27, HSP60, HSP70, IGF-I, IGF-II, Survivin, TNF-β, TRAILR-3, TRAILR-4 and XIAP, and the upregulation of pro-apoptosis protein Caspase 3 (Supplementary Figure 2). Meanwhile, we also evaluated the cell cycle distribution of cells with or without GSG2 knockdown, which clarified the significant decrease of cells in S phase with the concomitant increase of cells in G2 phase (P < 0.001, Figure 2E). Otherwise, the motility of bladder cancer cells was also restrained when treated with shGSG2 for GSG2 depletion, as presented by wound-healing (P < 0.05 for EJ, P < 0.01 for T24 cells, Figure 2F) and Transwell assays (P < 0.001, Figure 2G). Altogether, the *in vitro* studies illustrated the essential role of GSG2 in the development and progression of bladder cancer.

### Knockdown of GSG2 inhibited tumor growth of bladder cancer *in vivo*

**Figure 3:**
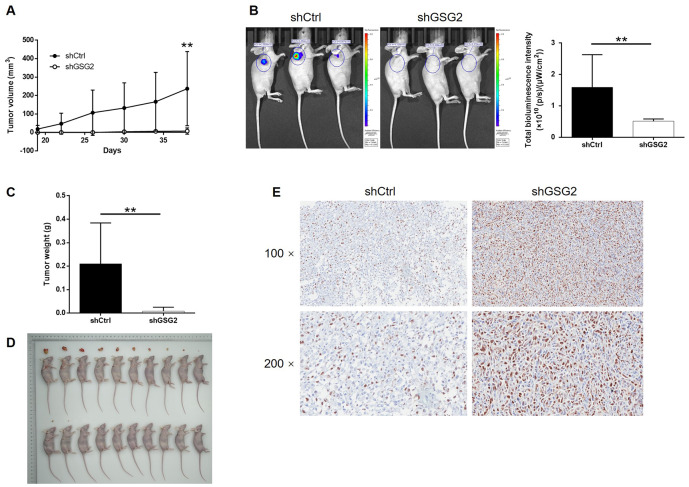
**GSG2 knockdown inhibited tumor growth of bladder cancer *in vivo*.** (**A**) The volume of tumors was measured and calculated throughout the culture of mice models. (**B**) The bioluminescent imaging of mice models was performed before the sacrifice of mice models. (**C**) The weight of tumors was measured after the removal of tumors from the mice models. (**D**) The photos of tumors removed from mice models were collected after the sacrifice of mice. (**E**) The expression of Ki-67 in the removed tumor tissues was detected by IHC. The figures are representative data from at least three independent experiments. The data were expressed as mean ± SD (n ≥ 3), *P<0.05, **P<0.01, ***P<0.001.

The influence of GSG2 depletion on bladder cancer tumor growth *in vivo* was subsequently investigated by using a xenograft model constructed with luciferase receptor containing cells. Tumor volume, bioluminescent intensity and tumor weight were evaluated as the representations of tumor development, all of which indicated the suppressed growth of tumors formed by cells with GSG2 knockdown (Figure 3A–3D). We also observed that the level of Ki-67, the typical biomarker for proliferation activity, was much higher in shCtrl group (Figure 3E). Collectively, knockdown of GSG2 was capable of inhibiting tumor development of bladder cancer *in vivo*.

### GSG2 may regulate bladder cancer development through targeting KIF15

**Figure 4:**
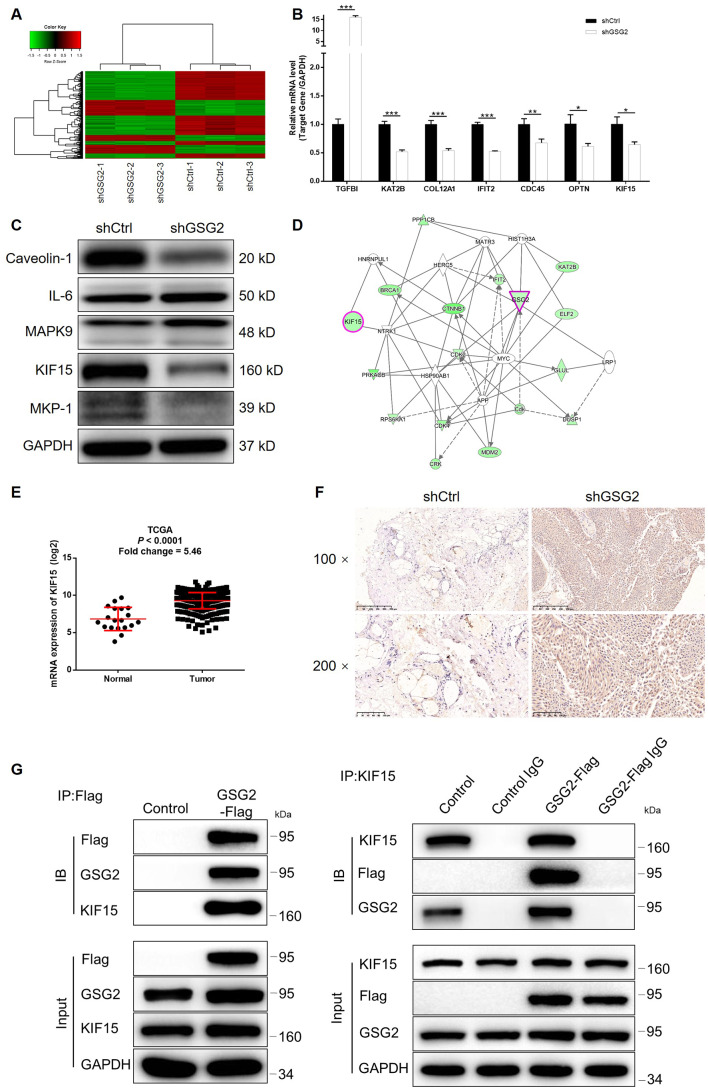
**KIF15 was identified as the downstream of GSG2 by RNA-sequencing.** (**A**) RNA-sequencing (3 v 3) was performed to identify differentially expressed genes in T24 cells with or without GSG2 knockdown. (**B**, **C**) The mRNA and protein levels of several top significant DEGs in shGSG2 group identified by RNA sequencing further detected in T24 cells were detected by qPCR (**B**) and western blotting (**C**), respectively. (**D**) The IPA analysis was performed to produce the GSG2-related interaction network. (**E**) The data mining of TCGA database showed the upregulation of KIF15 expression in bladder cancer tissues. (**F**) The expression of KIF15 in bladder cancer and normal tissues was detected by IHC. (**G**) The direct interaction between GSG2 and KIF15 was proved by co-immunoprecipitation. The data were expressed as mean ± SD (n ≥ 3), *P<0.05, **P<0.01, ***P<0.001.

Given the generally clear function of GSG2 in bladder cancer progression, we further utilized a microarray analysis based on T24 cells, in which GSG2 was silenced. According to the follow-up analysis of the sequencing data, 775 genes were found to be differentially expressed between shCtrl and shGSG2 groups, among which 273 were upregulated and 502 were downregulated (Figure 4A, Supplementary Figure 3A and 3B). Meanwhile, the enrichment of all differentially expressed genes (DEGs) in canonical signaling pathway or IPA disease and function was analyzed (Supplementary Figure 3C and 3D). Based on above results and our plan to seek for tumor promotor, several downregulated DEGs in the most enriched pathway or function were selected and focused. qPCR and western blotting were performed to verify the downregulated expression of the candidates in T24 cells with GSG2 depletion (Figure 4B, 4C and Supplementary Figure 3E). Moreover, taking KIF15, MAPK9 and TGFBI for example, subsequent screening showed that only knockdown of KIF15 by shKIF15-00141 could significantly inhibit cell proliferation while promote cell apoptosis (Supplementary Figure 4). Further through constructing molecular interaction network centered at GSG2, KIF15 was also identified as the most promising target of GSG2 (Figure 4D). The potential role of KIF15 in bladder cancer was next investigated by data mining of TCGA database and IHC analysis of clinical specimens, both of which suggested its upregulation in bladder cancer (Figure 4E and 4F). Consistently, the endogenous expression of KIF15 in bladder cancer cell lines was found to be significantly higher than that in HCV29 cell line as detected by qPCR (Supplementary Figure 5A). More importantly, co-immunoprecipitation assay indicated the direct interaction between GSG2 and KIF15 in T24 cells, paving a path for further investigating the regulatory mechanism (Figure 4G).

### Knockdown of KIF15 impaired the promotion of bladder cancer by GSG2 overexpression

**Figure 5:**
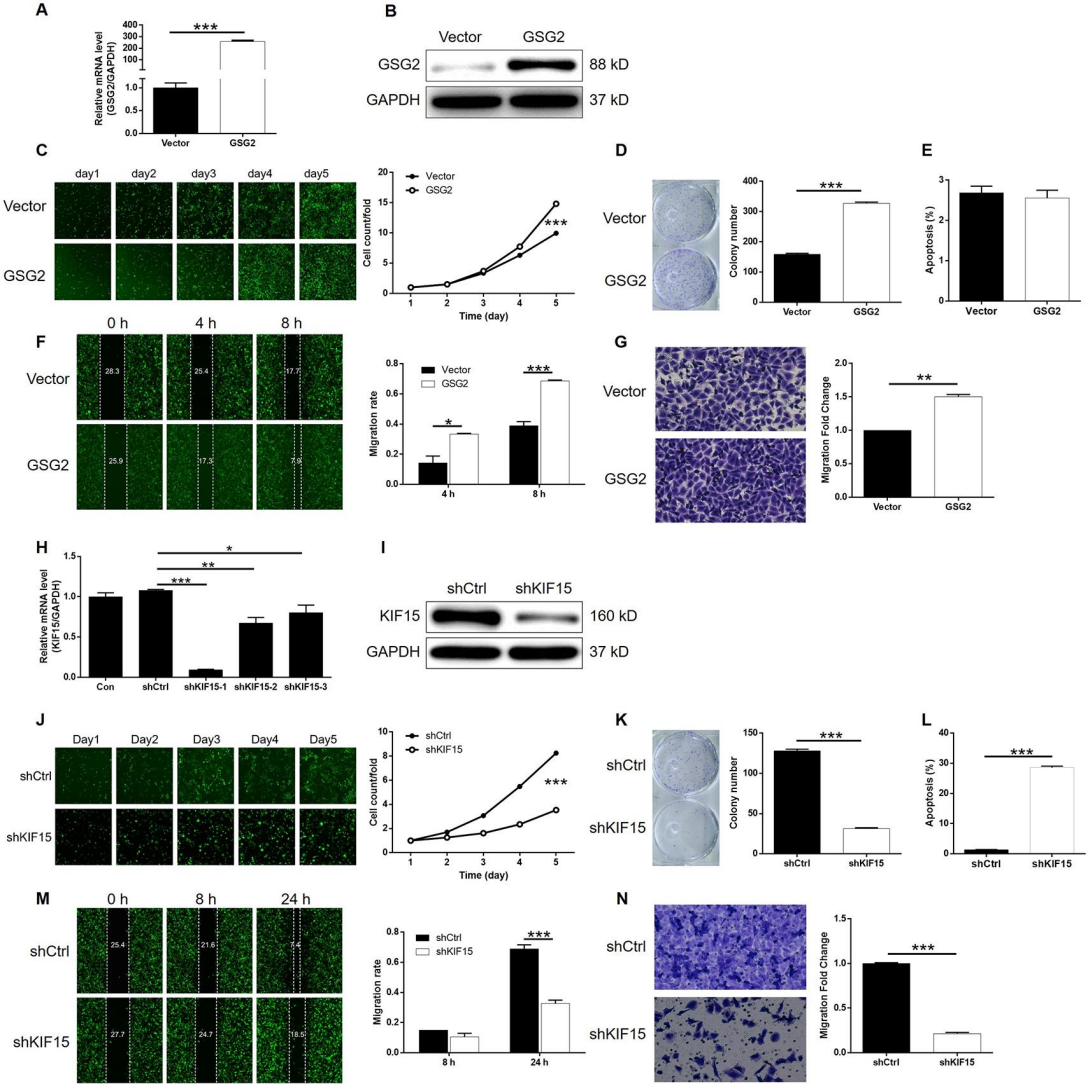
**Overexpression of GSG2 promoted bladder cancer and knockdown of KIF15 inhibited bladder cancer.** (**A**) The efficiency of GSG2 overexpression in T24 cells was evaluated by qPCR. (**B**) The successful overexpression of GSG2 in T24 cells was confirmed by western blotting. (**C**) Celigo cell counting assay was performed to examine the effects of GSG2 overexpression on cell proliferation of T24 cells. (**D**) The ability of T24 cells with or without GSG2 overexpression to form colonies was examined by colony formation assay. (**E**) Flow cytometry was performed to detect cell apoptosis of T24 cells with or without GSG2 overexpression. (**F**, **G**) The effects of GSG2 overexpression on cell migration of T24 cells were evaluated by wound-healing (**F**) and Transwell (**G**) assays. (**H**) The efficiency of KIF15 knockdown in T24 cells was evaluated by qPCR. (**I**) The successful knockdown of KIF15 in T24 cells was confirmed by western blotting. (**J**) Celigo cell counting assay was performed to examine the effects of KIF15 knockdown on cell proliferation of T24 cells. (**K**) The ability of T24 cells with or without KIF15 knockdown to form colonies was examined by colony formation assay. (**L**) Flow cytometry was performed to detect cell apoptosis of T24 cells with or without KIF15 knockdown. (**M**, **N**) The effects of KIF15 knockdown on cell migration of T24 cells were evaluated by wound-healing (**M**) and Transwell (**N**) assays. The figures are representative data from at least three independent experiments. The data were expressed as mean ± SD (n ≥ 3), *P<0.05, **P<0.01, ***P<0.001.

**Figure 6:**
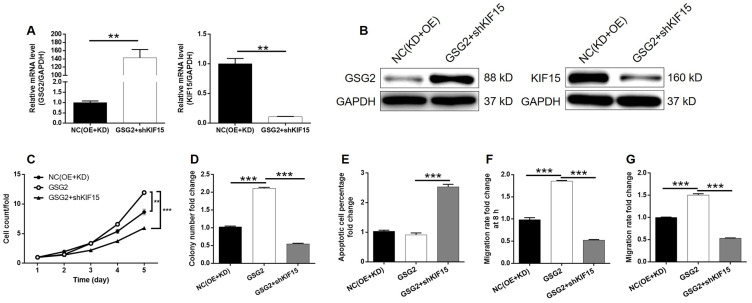
**KIF15 knockdown alleviated the promotion of bladder cancer by GSG2 overexpression.** (**A**, **B**) The mRNA and protein levels of GSG2 and KIF15 in T24 cells in NC(OE+KD) and GSG2+shKIF15 groups were detected by qPCR (**A**) and western blotting (**B**), respectively. (**C**) KIF15 knockdown reversed the promotion of cell proliferation of T24 cells by GSG2 overexpression. (**D**) KIF15 knockdown attenuate the effects of GSG2 overexpression on colony formation ability of T24 cells. (**E**) The slightly inhibited cell apoptosis of T24 cells by GSG2 overexpression was reversed by KIF15 knockdown. (**F**, **G**) The GSG2 overexpression induced promotion of cell migration of T24 cells detected by wound-healing (**D**) and Transwell (**E**) assays was alleviated by KIF15 knockdown. All data were collected from at least three independent experiments and were normalized to corresponding negative control for facilitating comparison. The figures are representative data from at least three independent experiments. The data were expressed as mean ± SD (n ≥ 3), *P<0.05, **P<0.01, ***P<0.001.

Although the interaction between GSG2 and KIF15 has been elucidated, their synergistic functions in bladder basically low spontaneous apoptosis of T24 cells cancer still needed to be investigated. For this purpose, T24 cell models with mere GSG2 overexpression, mere KIF15 depletion and concurrent GSG2 overexpression and KIF15 depletion were prepared, verified (Supplementary Figure 5B–5D) and used in the following functional experiments. First of all, several pieces of evidence were obtained by Celigo cell counting assay and colony formation experiments to show the promotion of bladder cancer by GSG2 overexpression (Figure 5A–5D). Notably, we failed to observe he anticipated inhibition of cell apoptosis by GSG2 overexpression, which may be attributed to the (Figure 5E). Not surprisingly, cell migration property visualized by wound-healing and Transwell assays was also enhanced in GSG2 overexpressed T24 cells (Figure 5F and 5G). In contrast, significant suppression of cell growth and cell motility, as well as promotion of cell apoptosis was observed in KIF15 deficient cells (Figure 5H–5N). Moreover, the results of the detections on GSG2+shKIF15 cell model, with practical upregulation of GSG2 and downregulation of KIF15 (Figure 6A, 6B), demonstrated that KIF15 knockdown could observably rescue the effects of GSG2 overexpression on cell proliferation, colony formation, cell apoptosis and cell migration (Figure 6C–6G). Therefore, it was functionally illustrated that KIF15 may be a target of GSG2 in the regulation of bladder cancer.

## DISCUSSION

GSG2 kinase is an atypical member of eukaryotic protein kinases (ePK) which structurally diverges from most ePK’s [13, 14]. The functional research revealed that GSG2 could phosphorylate the highly conserved threonine 3 site of histone H3 *in vitro* [15], which is an essential process in mitotic cells. Meanwhile, the knockdown of GSG2 could induce chromosome dislocation in metaphase while its overexpression delays progression through early mitosis [15, 16]. Moreover, Wang et al. reported that the GSG2-induced phosphorylation of histone H3 threonine 3 is an essential factor in accumulation of chromosomal passenger complex (CPC) at centromeres thus regulating selected targets of Aurora B during mitosis, which could be blocked by GSG2 depletion [17]. Zhou et al. further demonstrate that Polo-like kinase-1 (Plk1) triggered initial activation of GSG2 in early mitosis, thus regulating CPC recruitment during mitosis [18]. Moreover, considering the essential role of phosphorylation of histone H3 at Thr3 residue in mitosis progression which is only regulated by GSG2, the inhibition of GSG2 has been identified as a promising target for the design of small molecule inhibitors as anticancer drugs [19]. For example, Huertas et al. identified CHR-6494 as a first-in-class inhibitor for GSG2 which exhibited a wide spectrum of anticancer effects [19]. Kim et al. found that Coumestrol possessed epigenetic suppression cell proliferation of skin melanoma, lung cancer and colon cancer cells through directly targeting GSG2 [11]. Although various inhibitors of GSG2 has been proved to display anticancer activity, the role of GSG2 itself in the development and progression of human cancers including bladder cancer is still rarely reported and remains largely unknown.

In this study, we found the upregulation of GSG2 in tumor tissues and cancer cell lines of bladder cancer compared with normal tissues and normal cell line, which is in agreement with the previously reported tumor promotion effects of GSG2. Also, high GSG2 expression was analyzed to be significantly correlated with more advanced malignant grade and poorer prognosis of patients with bladder cancer. Subsequently, the loss-of-function investigation revealed that GSG2 knockdown inhibited bladder cancer development *in vitro* and *in vivo*, which may be derived from the regulation of cell apoptosis and cell cycle distribution. Through the analysis of an apoptosis antibody array, we identified the downregulated anti-apoptosis proteins and the upregulated pro-apoptosis proteins, by which may GSG2 regulates cell apoptosis of bladder cancer. On the other hand, the inhibitory ability of GSG2 knockdown on cell motility of bladder cancer was also confirmed in our study. Moreover, the gain-of-function research revealed the promotion effects of GSG2 on bladder cancer development. As the next step, the regulatory mechanism of GSG2 was explored, identifying KIF15 as a promising downstream target of GSG2.

Kinesin protein superfamily (KIF) could use the energy released by adenosine triphosphatase hydrolysis to drive the molecules carried by it to move along the positive pole of microtubule filaments to carry out intracellular substances (such as information molecules, vesicles, organelles, chromosomes, protein complexes) and mRNA transport, regulating signaling pathways, and thus executing their biological functions [20]. At present, the kinesin superfamily has a total of 45 members and is divided into 14 subfamilies named kinesin 1 ~ kinesin 14 [21]. Previous studies have revealed that KIFs were related with neurodegenerative diseases such as Alzheimer’s disease [22] and Huntington’s disease [23], diabetes [23] and nephropathy [25]. Recently, their functions in human cancers have attracted more and more attention. Lucanus et al. summarized the various members of kinesin superfamily that are involved in the development and progression of breast cancer or related to the prognosis of breast cancer patients [26]. They also indicated that some KIFs has presented their potential as therapeutic target in the treatment of breast cancer [26].

Similar study was also performed and reported by Song et al., showing the relationship between high expression of KIF15, KIF20A, KIF23, KIF2C, KIF4A and poor prognosis [27]. Moreover, KIF11 was identified as an oncogene in breast cancer through prompting cell proliferation, colony formation, cell migration while inhibiting cell apoptosis [28]. Li et al. reported that KIF23 is able to promote the development of gastric cancer through stimulating the expression of Ki-67 and PCNA, thus accelerating cell proliferation [29]. All these results potentiate the potential role of KIFs in the promotion of cancer development and progression. KIF15, also named kinesin-12, is a microtubule-dependent plus-end-directed motor protein which was best known for its role in mitosis. McHugh et al. found that, in the center spindle, KIF15 may act as a mechanical ratchet, supporting spindle extension, but resisting spindle compression [29]. The functions of KIF15 in human cancers were also investigated to some extent. For example, Sheng et al. reported that KIF15 expression in triple-negative breast cancer (TNBC) was negatively related with patients’ prognosis, knockdown of which inhibited TNBC cell proliferation [31]. High expression of KIF15 in melanoma was also observed by Yu’s group, whose report illuminated the positive role of KIF15 in the tumorigenicity of melanoma [32]. Moreover, Zhao’s group identified KIF15 as a upstream regulator of MEK-ERK signaling pathway, by which may KIF15 promoted pancreatic cancer [33]. Herein, as a potential downstream target of GSG2, KIF15 was found to possess similar effects with GSG2 on bladder cancer development. Knockdown of KIF15 could significantly inhibit cell proliferation and cell migration of bladder cancer, while promoting cell apoptosis. More importantly, KIF15 knockdown could alleviated the promotion of bladder cancer by GSG2 overexpression.

In conclusion, we found the upregulated expression of GSG2 and KIF15 in tumor tissues and cancer cell lines of bladder cancer. Both GSG2 and KIF15 could act as tumor promotor in the development and progression of bladder cancer, through promoting cell proliferation, colony formation, cell migration and suppressing cell apoptosis. More importantly, experimental evidence proved that GSG2 may regulate bladder cancer through its downstream target KIF15. Therefore, all the results identified GSG2 as a potential therapeutic target for bladder cancer treatment.

## MATERIALS AND METHODS

### Cell culture

HCV29, RT4, T24, EJ and J82 cell lines were purchased from BeNa Technology (Hangzhou, Zhejiang, China). RT4 cells were cultured in McCOY’s 5A (Sigma, St Louis, MO, USA) medium. HCV-29, EJ and T24 were cultured in 90% RPMI-1640 with 10% FBS additives, and J82 cells were cultured in MEM medium (Gibco, Rockville, MD, USA). All culture medium was changed every 3 days.

### Immunohistochemistry (IHC)

The formalin-fixed, paraffin-embedded tissue microarray of bladder cancer were purchased from Shanghai Outdo Biotech Company (Cat. # XT12-065, China). Bladder cancer patients’ information and related data were collected and written informed consent was provided by each patient. Our study protocol was approved by Ethics committee of Jinling Hospital. Xylene were used for paraffin section dewaxing and 100% alcohol for hydration. PBS-H_2_O_2_ with 0.1% Tween 20 were added. Citric acid buffer was added for antigen retrieval with heating at 120°C for 20 min. After washing, the slides were incubated with primary antibodies overnight at 4°C and then incubated with second antibody for 2 h at room temperature. DAB color was developed with diaminobenzene for 10 min and then counterstained with hematoxylin. The slides were dehydrated, and exanimated with 200× and 400× objective microscopic. Appropriate positive and negative controls were tested in parallel.

IHC scoring standard for GSG2 was graded as 0 (negative), 1 (weak), 2 (positive ++) and 3 (positive +++). The staining extent was graded as 0 (0%), 1 (1-25%), 2 (26-50%), 3 (51-75%), or 4 (76-100%). The staining intensity varied from weak to strong. Specimens were classified into negative (0), positive (1-4), ++ positive (5-8), or +++ positive (9-12), based on the sum of the staining intensity and staining extent scores.

### Lentivirus plasmid construction

The negative control and lentivirus containing the GSG2 (GSG2-Flag) overexpression and knockdown sequences (5’-CCACAGGACAATGCTGAACTT-3’ for GSG2; 5’-GCTGAAGTGAAGAGGCTCAAA-3’, 5’-AGGCAGCTAGAATTGGAATCA-3’, 5’-AAGCTCAGAAAGAGCCATGTT-3’ for KIF15) were purchased from Shanghai Yibeirui Biomedical Science and Technology Co., Ltd. (Shanghai, China). The overexpression sequences and shRNAs were subsequently cloned into pGCSIL-green fluorescent protein lentivirual vector BR-V-108 to generate recombined lentiviral vectors. Lentiviral vectors and packaging vectors were transfected into 293T cells using Lipofectamine 2000 (Invitrogen, New York, CA, USA) according to manufacturer’s instructions. Lentiviral particles were purified using ultracentrifugation, and an endpoint dilution assay was performed to determine the titer of the lentiviruses.

### Lentivirus transfection

EJ and T24 cells were cultured in 6-well plates at a density of 4.0×10[5] cells/well, infected with lentivirus (4×10^8^ TU/mL × 2.5 μL) or negative control (8×10^8^ TU/mL×1.25 μL) lentivirus. The cells were observed under a fluorescence microscope (MicroPublisher 3.3RTV; Olympus, Tokyo, Japan) after 72 h. Following 5 days of transfection, the knockdown efficiency of shGSG2 and shKIF15 vectors was investigated *via* qPCR analysis.

### Cell apoptosis and cell cycle

EJ and T24 cells were seeded in 6-well plates (1 × 10^3^ cells/mL) for 5 days. 5 μL Annexin V-APC was added for staining 10-15 min at room temperature in the dark. The percentage of cells phases was measured using FACScan (Becton Dickinson, Franklin Lakes, NJ, USA) to assess the apoptotic rate, and results were analyzed. All cells were cultured in 37°C with 5% CO_2_.

For cell cycle assay, cells were stained with 1 mL cell staining solution (40×PI, 2 mg/mL: 100×RNase, 10 mg/mL: 1×PBS =25:10:1000) for 30 min. FACScan and FlowJo 7.6.1 (Ashland, OR, USA) was used for analyze. The percentage of the cells in G0-G1, S, and G2-M phase were counted and compared. Each experiment was repeated three times.

### Celigo cell counting assay

Lentivirus-infected T24 cells and negative control cells were seeded at a 96-well plate with 2,000 cells per well for culturing. Each group set three wells. The plate was continuously detected by Celigo (Nexcelom, Lawrence, MA, USA) for 5 days at the same time. Cell proliferation rate was analyzed.

### qPCR

Total RNA was isolated from EJ and T24 cells using TRIzol^®^ reagent (Thermo Fisher Scientific, Waltham, MA, USA) according to the manufacturer’s instructions. The purity and integrity of RNA was assessed by Nanodrop 2000/2000C spectrophotometry (Thermo Fisher Scientific, Waltham, MA, USA). Total RNA (1 μg) was reversely transcribed to high-quality cDNA with HiScript Q RT SuperMix for qPCR (+gDNA wiper) (Vazyme, Nangjing, Jiangsu, China) according to the manufacturer's protocol. qPCR was performed using AceQ qPCR SYBR Green Master Mix (Vazyme, Nangjing, Jiangsu, China). GAPDH acted as an endogenous control. Quantification of gene expression was performed using the 2^-Δ ΔCt^ method. The primer sequences used showed in Supplementary Table 2.

### Western blotting (WB)

EJ and T24 cells were fully lysed with ice-cold RIPA lysis buffer and total proteins were extracted. Protein concentration was detected by a BCA Protein Assay Kit (HyClone-Pierce, Logan, UT, USA). 20 μg proteins in each lane were separated by 10% SDS-PAGE gel, and then transferred to a PVDF membrane. The membrane was blocked by 5% non-fat milk. Blocked membrane was incubated with primary antibodies at 4°C overnight and subsequently incubated with second antibodies. The antigen-antibody complexes were detected using ECL-PLUS/Kit (Amersham, Chicago, IL, USA). Antibodies used in Western Blot assay showed in Supplementary Table 3.

For co-immunoprecipitation assay, whole cell lysate was collected from T24 cells with or without GSG2-Flag overexpression and used for co-immunoprecipitation by anti-Flag or anti-KIF15. The immunocomplex was further detected by using anti-Flag, anti-GSG2 and anti-KIF15 antibodies.

### MTT assay

For MTT assay, EJ and T24 cells (2,500 cells/well) were seeded onto a 96-well plate. 20 μL MTT solution (5 mg/mL, GeneView, El Monte, CA, USA) was added to each well. 4 h later, 150 μL DMSO was added as well. Absorbance values at 490 nm were measured using a microplate reader (Tecan, Männedorf, Zürich, Switzerland) after 24, 48, 72, 96, and 120 h of growth, and 570 nm was the reference wavelength. Cell viability ratio was calculated (Cell viability (%) = optical density (OD) treated/OD control × 100%).

### Colony formation assay

Lentivirus-infected T24 cells were collected, digested and resuspended (2,500 cells/mL). For colony formation, 2 mL cell suspension was seeded in a 6-well plate and cultured 8 days, the culture medium was changed every 3 days. Colony photos were collected by fluorescence microscope (Olympus, Tokyo, Japan). Finally, cells were fixed with 4% paraformaldehyde and stained by Giemsa (Dingguo, Shanghai, China) and the number of colonies (> 50 cells/colony) was counted.

### Wound healing assay

EJ and T24 cells (3×10^4^/well) were seeded into 6-well dishes and grew 72 h. A line wound was made across the cell layer and cells were cultured in an incubator culture at 37°C with 5% CO_2_ for 8 h. Photograph were taken by fluorescence micrograph at 4 h and 8 h post wound making. Cell migration rate of each group was calculated based on the pictures.

### Transwell assay

100 μL EJ and T24 cell suspension (total 5 × 10^4^ cells) was loaded into the serum-free medium upper chamber of the Transwell (24-well, 8-mm pore, Corning, NY, USA). The lower chamber was filled with 600 μL culture medium containing 30% FBS for incubation 24 h at 37°C. The non-invading cells in the upper chamber were removed and the cells adhering to the membrane were fixed in 4% paraformaldehyde and stained with 0.1% crystal violet. MTT Values of Cell Number were measured by CellTiter 96 AQueous One Solution Cell Proliferation Assay (Promega, Heidelberg, Germany). 100x and 400x microscope pictures were collected and the transfer rates were calculated.

### RNA sequencing

Total RNA from GSG2 Gene knockdown cell model for microarray experiments was extracted by Trizol according to the manufacturer’s guidelines. RNA concentration and purity were quantified using a NanoDrop 2000 (Thremo Fisher Scientific, Waltham, MA, USA) and Agilent 2100 Bioanalyzer RNA Nano Chip (Agilent Technologies, Palo Alto, CA, USA). Human GeneChip primeview (Affymetrix, Santa Clara, CA, USA) was used for microarray processing to determine gene expression according to the manufacturer’s instructions. RNA sequencing data processing and analysis were performed with R studio. Limma package was used for cluster analysis and differential expression of genes assessing. Canonical pathways, diseases and functions, molecular and cellular processes that are significantly associated with differentially expressed genes (DEGs) in the data sets were determined using Ingenuity Pathway Analysis (IPA) software. |Z score| > 2 is considered to be significant.

### Human apoptosis antibody array

Total protons were isolated from shCtrl and shGSG2 T24 cells and the concentration of proteins were detected by a BCA Protein Assay Kit (HyClone-Pierce, Logan, UT, USA). Human Apoptosis Antibody Array (Cat # ab134001, Abcam, Cambridge, MA, USA) were used and biotin-conjugated anti-cytokines and HRP-Conjugated Streptavidin and chemiluminescent detection reagents were added. Spots on the array membrane were detect by CCD cameras for imaging.

### Mice xenograft model

Specific pathogen-free (SPF) 4-week-old, female BALB/c nude mice (Shanghai Lingchang Animal Research Institute, China) were randomly divided into shGSG2 and shCtrl group (5 mice in each group). 0.2 mL (6 × 10^7^ cells/mL) shGSG2 and shCtrl T24 cell suspensions were injected into the right back of each mouse. Mice weight and tumor length and width were recorded every 4 days for 6 weeks. The volume of tumors was estimated based on the measurement of length and width at 19, 22, 26, 30, 34 and 38 days post injection. Then all mice were anaesthetized by intraperitoneal injection of 0.7% sodium pentobarbital (10 μL/g) and the anaesthetized mice were placed under a Perkin Elmer IVIS Spectrum (Waltham, MA, USA) for *in vivo* bioluminescence imagine. Finally, all mice were sacrificed, and the tumors were removed for taking photos and weighting. All animal experiments performed in our study were approved by Ethics committee of Jinling Hospital.

### Ki-67 immunostaining assay

Mice tumor sections were fixed in 4% paraformaldehyde for 16 h. Paraffin embedded 5 μm sections were made for H&E and IHC staining. We added citric acid buffer for antigen retrieval at 120°C. Sections were blocked using PBS-H_2_O_2_ with 0.1% Tween 20. Ki-67 antibody was added for incubating at 4°C overnight and then secondary antibodies were added as well (Supplementary Table 3). DAB color was developed with diaminobenzene for 10 min and then counterstained with hematoxylin. Stained slides were pictured with a microscopic.

### Statistical analysis

Data in our study were expressed as percentages or Means ± SD. SPSS 18.0 software (Chicago, IL, USA) and GraphPad Prism 7.0 (La Jolla, CA, USA) were used. Student’s t-test was used while analysis the significant differences of two groups. For multiple groups, one-way analysis of variance test was used. Chi-squared test was applied for evaluating the GSG2 expression differences between tumor tissues and para-carcinoma tissues. Spearman rank correlation analysis and Mann-Whitney U analysis was used to evaluate the association between GSG2 expression and characteristics of bladder cancer patients. *P* < 0.05 was considered as significant.

## Supplementary Material

Supplementary Figures

Supplementary Tables
