# Supplementary material for: GSG2 (Haspin) promotes development and progression of bladder cancer through targeting KIF15 (Kinase-12)
Source: Aging (Albany NY). 2020 May 21;12(10):8858–79. doi: 10.18632/aging.103005 (PMC7288960; doi:10.18632/aging.103005)
Supplement: Supplementary Figures [file aging-12-103005-s002..pdf]

SUPPLEMENTARY FIGURES

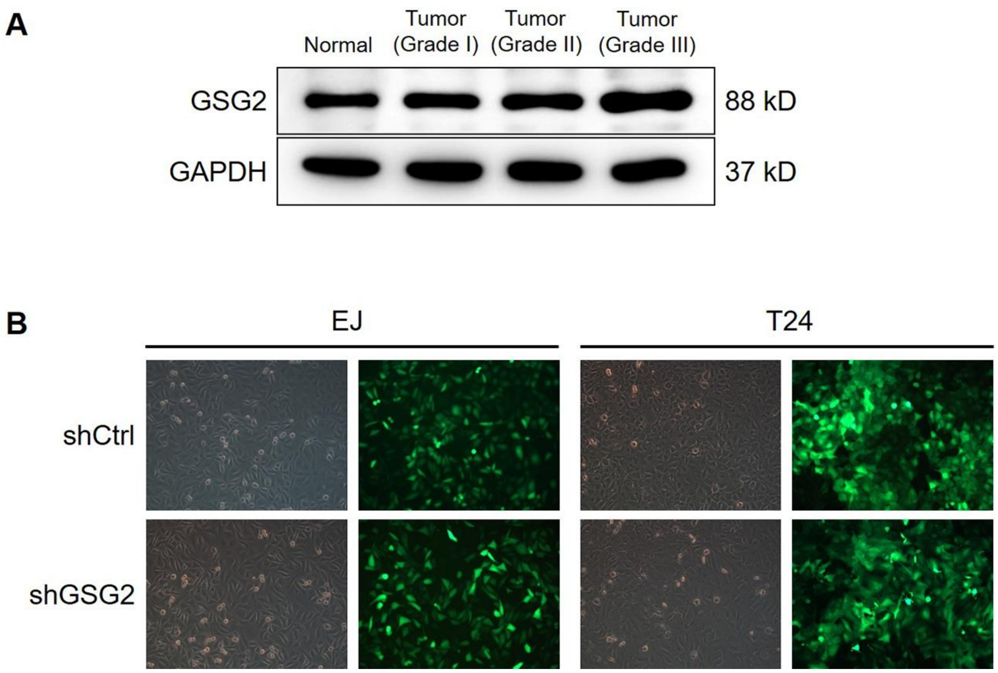

Supplementary Figure 1. (**A**) The protein level of GSG2 was detected in normal tissue and tumor tissues with different malignant grades. (**B**) The transfection efficiencies of shGSG2 and shCtrl of EJ and T24 cells were evaluated through observing fluorescence of GFP tag on lentivirus vector.

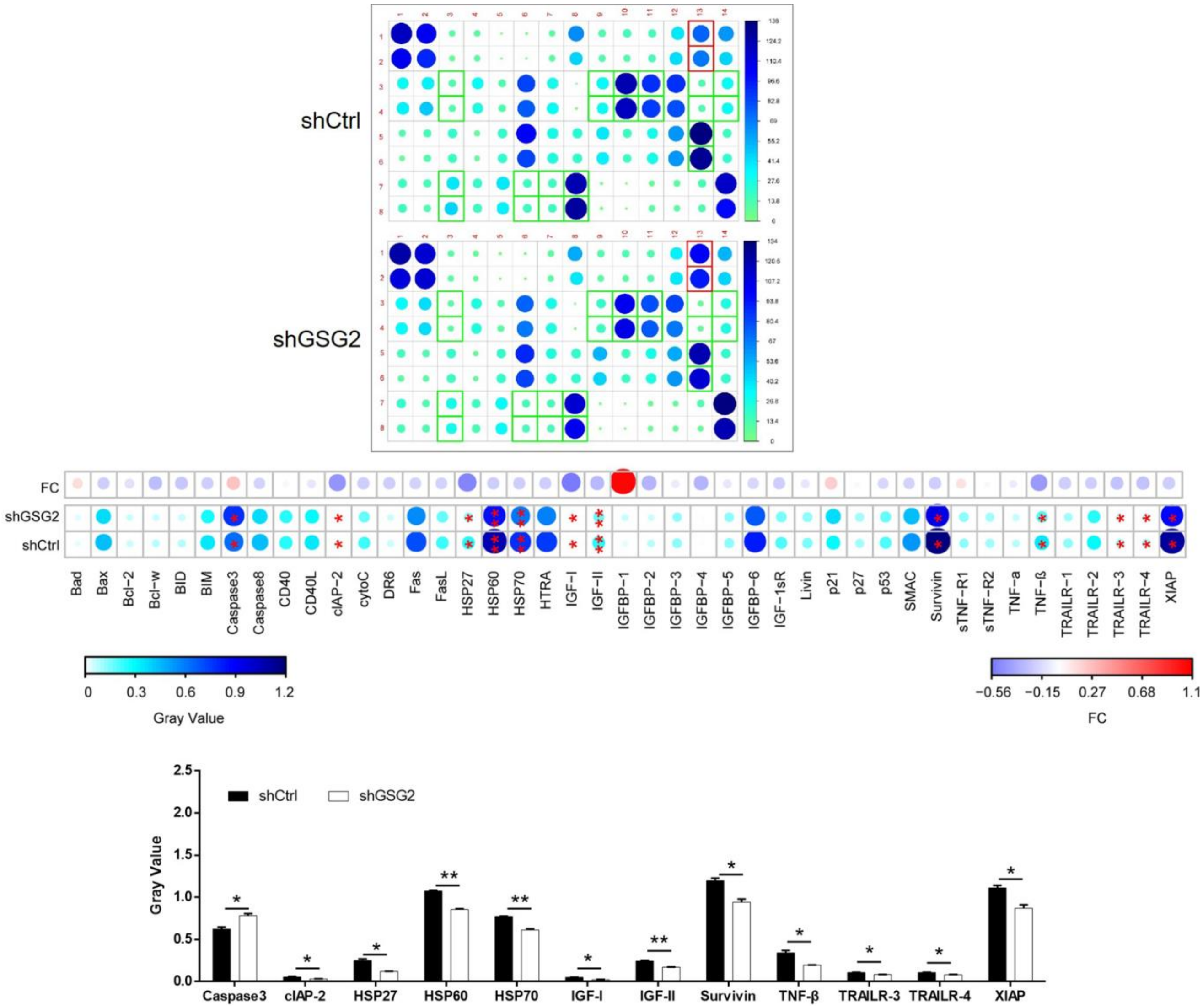

Supplementary Figure 2. Human apoptosis antibody array was utilized to illustrate the regulation of the expression of apoptosis related proteins by GSG2 knockdown.

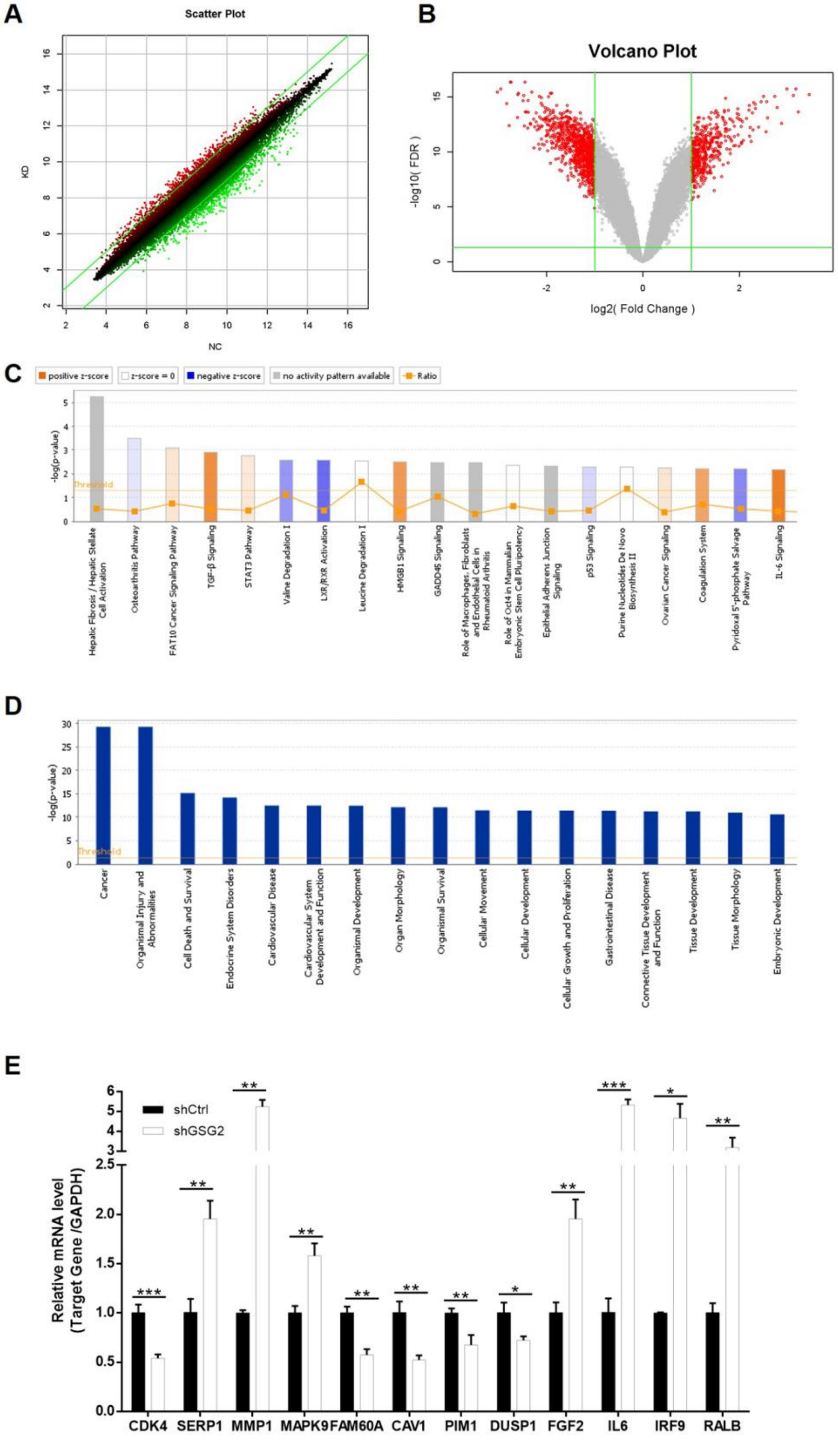

Supplementary Figure 3. (**A**) Scatter plot of RNA-sequencing (3 v 3) between T24 cells with or without GSG2 knockdown. (**B**) Volcano plot of RNA-sequencing (3 v 3) between T24 cells with or without GSG2 knockdown. (**C**) IPA analysis was performed to identify the enrichment of DEGs in canonical signaling pathways. (**D**) IPA analysis was performed to identify the enrichment of DEGs in IPA disease & function. (**E**) The mRNA levels of various DEGs in shGSG2 groups were verified in EJ cells through qPCR. The data were expressed as mean ± SD (n ≥ 3), **P*<0.05, ***P*<0.01, ****P*<0.001.

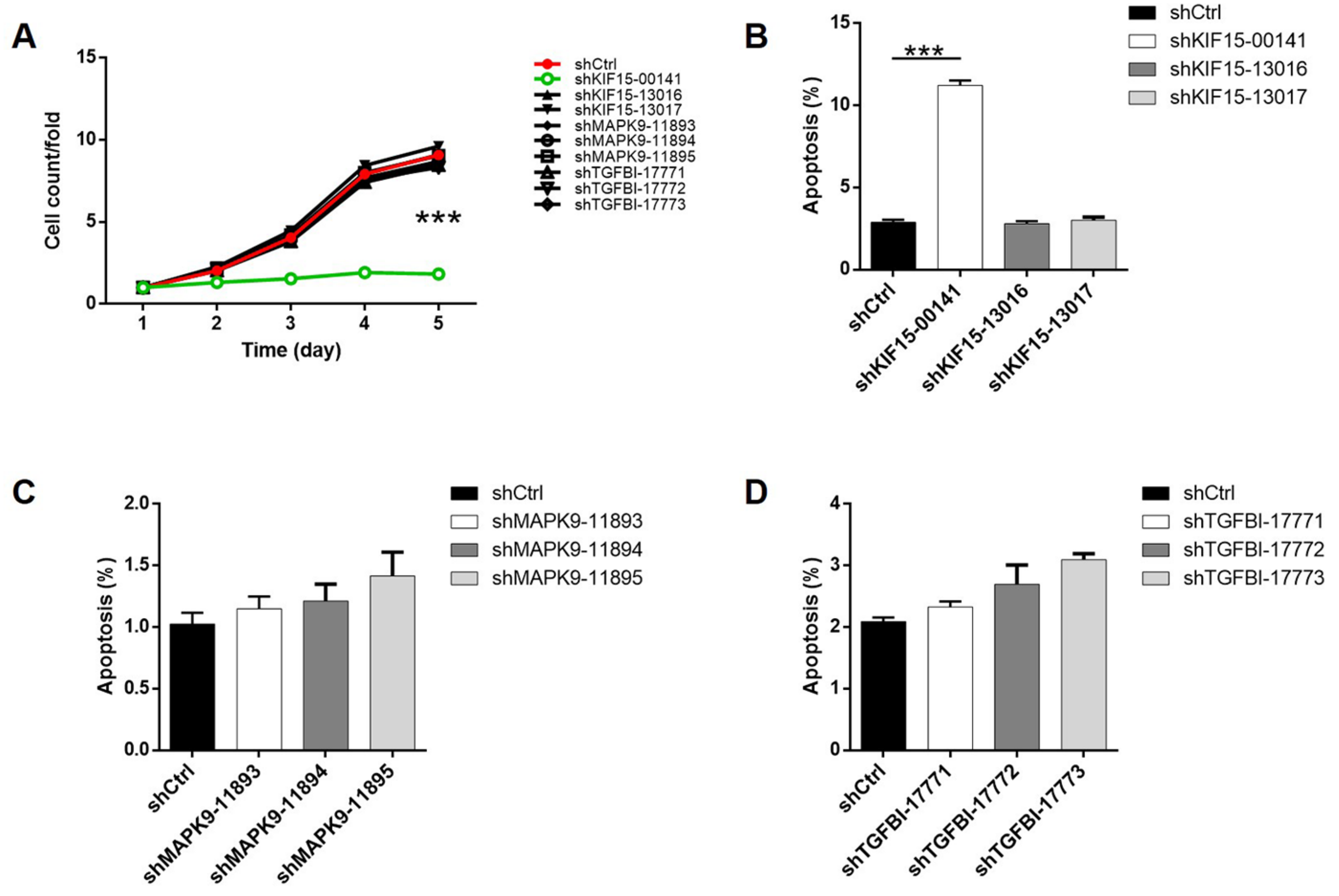

Supplementary Figure 4. (**A**) The effects of candidate gene knockdown on T24 cell proliferation were evaluated by celigo cell counting assay. (**B**–**D**) The effects of candidate gene knockdown on T24 cell apoptosis were assessed by flow cytometry. The data were expressed as mean ± SD (n ≥ 3), **P*<0.05, ***P*<0.01, ****P*<0.001.

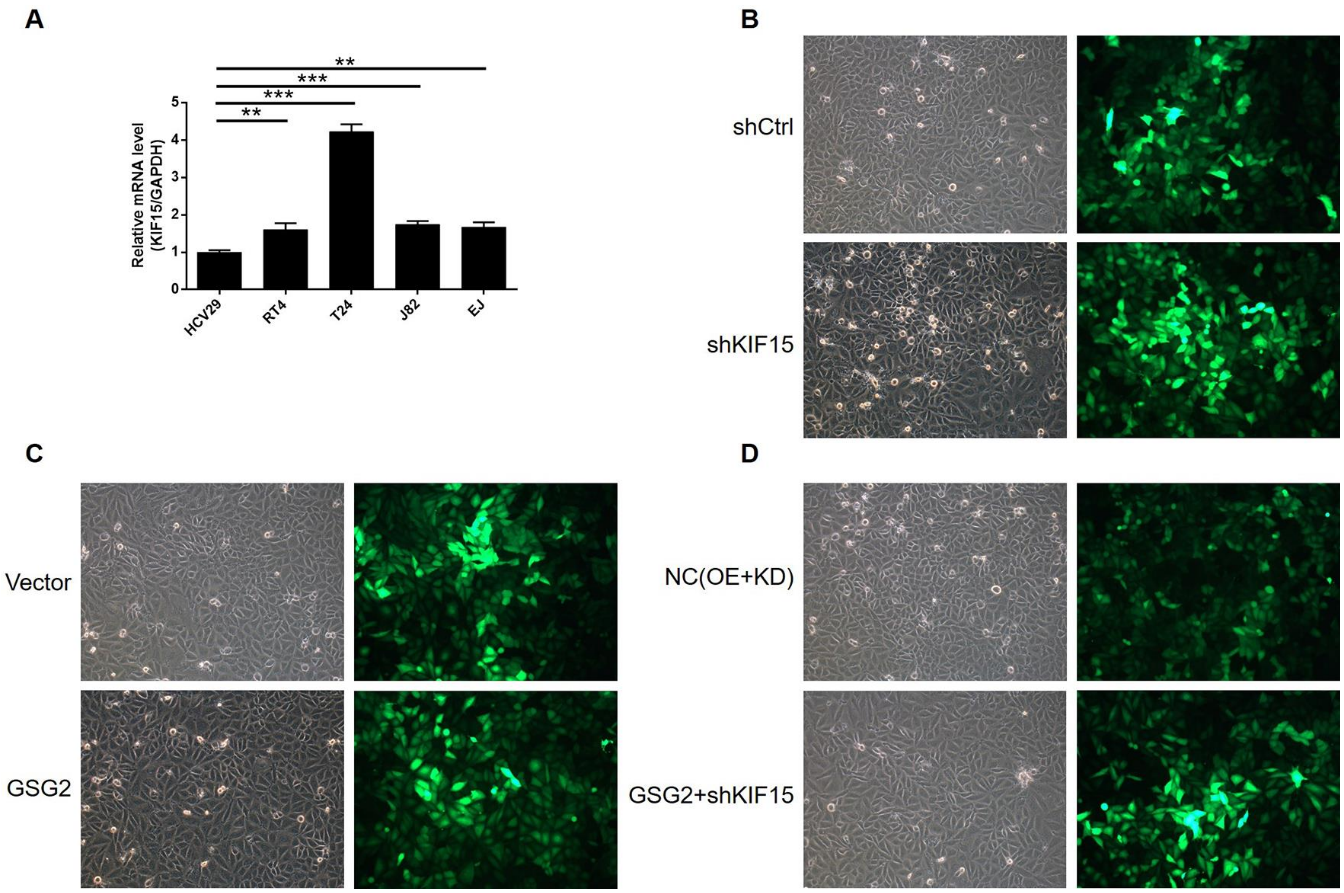

Supplementary Figure 5. (**A**) The endogenous expression of KIF15 in human bladder epithelial cell line HCV29 and bladder cancer cell lines including RT4, EJ, T24 and J82 was detected by qPCR. (**B**) The transfection efficiencies of shKIF15 shCtrl were evaluated through observing fluorescence of GFP tag on lentivirus vector. (**C**) The transfection efficiencies of GSG2 overexpression plasmids and the negative control plasmids (Vector) were evaluated through observing fluorescence of GFP tag on lentivirus vector. (**D**) The transfection efficiencies of GSG2+shKIF15 plasmids for simultaneous overexpressing GSG2 and silencing KIF15 and the negative control (NC(OE+KD)) were evaluated through observing fluorescence of GFP tag on lentivirus vector. The data were expressed as mean ± SD (n ≥ 3), **P*<0.05, ***P*<0.01.
