# Supplementary material for: GSG2 (Haspin) promotes development and progression of bladder cancer through targeting KIF15 (Kinase-12)
Source: Aging (Albany NY). 2020 May 21;12(10):8858–79. doi: 10.18632/aging.103005 (PMC7288960; doi:10.18632/aging.103005)
Supplement: Supplementary Tables [file aging-12-103005-s001..pdf]

SUPPLEMENTARY TABLES

Supplementary Table 1. Relationship between GSG2 expression and tumor characteristics in patients with bladder cancer analyzed by spearman rank correlation analysis.

Tumor characteristics	index	
Grade	Pearson correlation	0.398
	Significance (two tailed)	0.002**
	n	56

Supplementary Table 2. Primers used in qPCR.

Gene	Forward primer sequence (5'-3')	Reverse primer sequence (5'-3')
GAPDH	TGACTTCAACAGCGACACCCA	CACCCTGTTGCTGTAGCCAAA
GSG2	GGAAGGGGTGTTTGGCGAAGT	TGAGGAGCAAGGGAGGGTAAG
TGFBI	TGCTCCCACAAATGAAGCCT	GCCTCCGCTAACCAGGATTT
KAT2B	GAATCGCCGTGAAGAAAGCG	TGGGTGAGGGGTTAGGGTTT
COL12A1	GTGAAACCAACAGAAGCCCCTAC	TGGCCCCTTTGCATACATCC
IFIT2	AATAGGACACGCTGTGGCTC	AGGCTGGCAAGAATGGAACA
CDC45	TTTGGGAGGGCGTTTGAGA	GAGGGAAATAAGTGCGTCCAG
OPTN	AGACCTGTTGGGCATCGTGT	TTACTGACCCTTCTGCTTCTCC
FGF2	AGCGACCCTCACATCAAGCT	GCCAGGTAACGGTTAGCACA
CDK4	CTACCAGATGGCACTTACACCC	GCAAAGATACAGCCAACACTCC
SERP1	GAGAAGCACAGCAAGAACATCA	CCAATAACCAGGGTCCTACAGAC
MMP1	ACGATTCGGGGAGAAGTGATG	TGTCGGCAAATTCGTAAGCAG
MAPK9	CTCTGCGTCACCCATACATCA	TCTTTCTTCCAACTGGGCATC
FAM60A	CAAGCCAAAGATGTACCGAAGT	CATTGCAGATGTCTCCTGAACGA
CAV1	TCTGGGGCATTTACTTCGC	GATGGAATAGACACGGCTGATG
PIM1	TTTTCTTCAGGCAGAGGGTCT	GGAGGTGGATCTCAGCAGTTT
DUSP1	ACCACCACCGTGTTCAACTT	AGAGGTCGTAATGGGGCTCT
KIF15	CTCTCACAGTTGAATGTCCTTG	CTCCTTGTCAGCAGAATGAAG
IL6	AAAGGCTGTGCTCTTGGTGA	TGGGACTCCTGGGAATACTG
IRF9	GAGCCACAGGAAGTTACAGACAC	GCCCGTTGTAGATGAAGGTGA
RALB	ACTGCTCGTCGTGGGAAACA	CCACTCTTCGGCTTTACTCCTG

Supplementary Table 3. Antibodies used in western blotting and IHC.

Primary antibodies	Dilution in WB	Source species	Company	Catalog No.
GSG2	1:1000	Rabbit	abcam	ab21686
GAPDH	1:3000	Rabbit	Bioworld	AP0063
Caveolin-1	1:1000	Rabbit	Abcam	ab2910
IL-6	1:500	Mouse	Abcam	ab9324
JNK2	1:1000	Rabbit	Abcam	ab76125
KIF15	1:1000	Rabbit	Fine test	FNab04551
MKP-1	1:500	Rabbit	Abcam	ab138265
GAPDH	1:3000	Rabbit	Bioworld	AP0063
**Primary antibodies**	**Dilution in IHC**	**Source species**	**Company**	**Catalog No.**
GSG2	1:200	Rabbit	Bioss	bs-15413R
KIF15	1:50	Rabbit	Fine test	FNab04551
Ki-67	1:200	Rabbit	Abcam	ab16667
Secondary antibody	Dilution		Company	Catalog No.
HRP Goat Anti-Rabbit IgG (WB)	1:3000		Beyotime	A0208
HRP Goat Anti-Mouse IgG (WB)	1:3000		Beyotime	A0216
HRP Goat Anti-Rabbit IgG (IHC)	1:200		Abcam	Ab111909
